# Supplementary figures and images for: Population dynamics of free-roaming dogs in two European regions and implications for population control
Source: PLoS One. 2022 Sep 9;17(9):e0266636. doi: 10.1371/journal.pone.0266636 (PMC9462782; doi:10.1371/journal.pone.0266636)

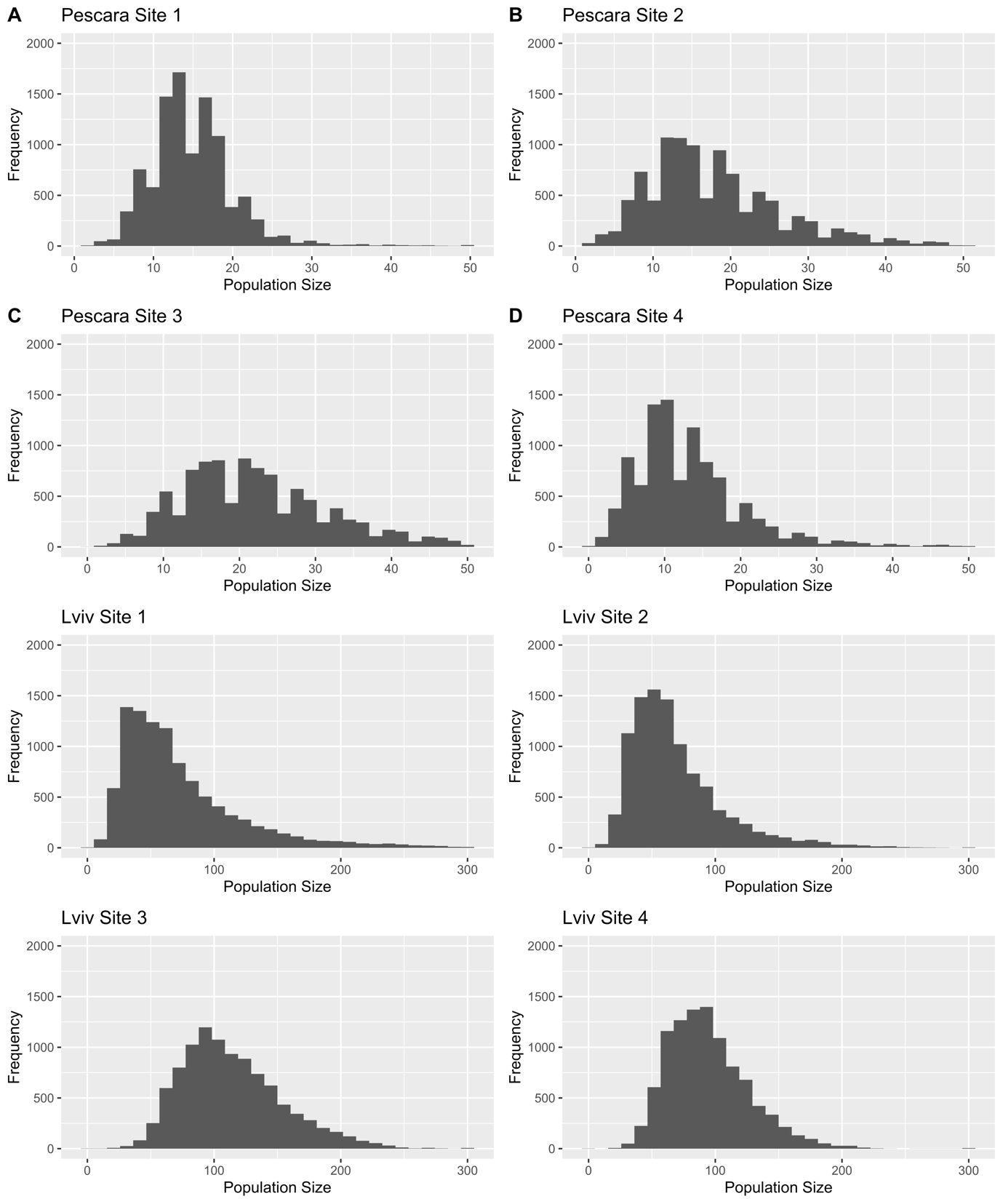

Supplement: S1 Fig — (TIF) [file pone.0266636.s001.tif]

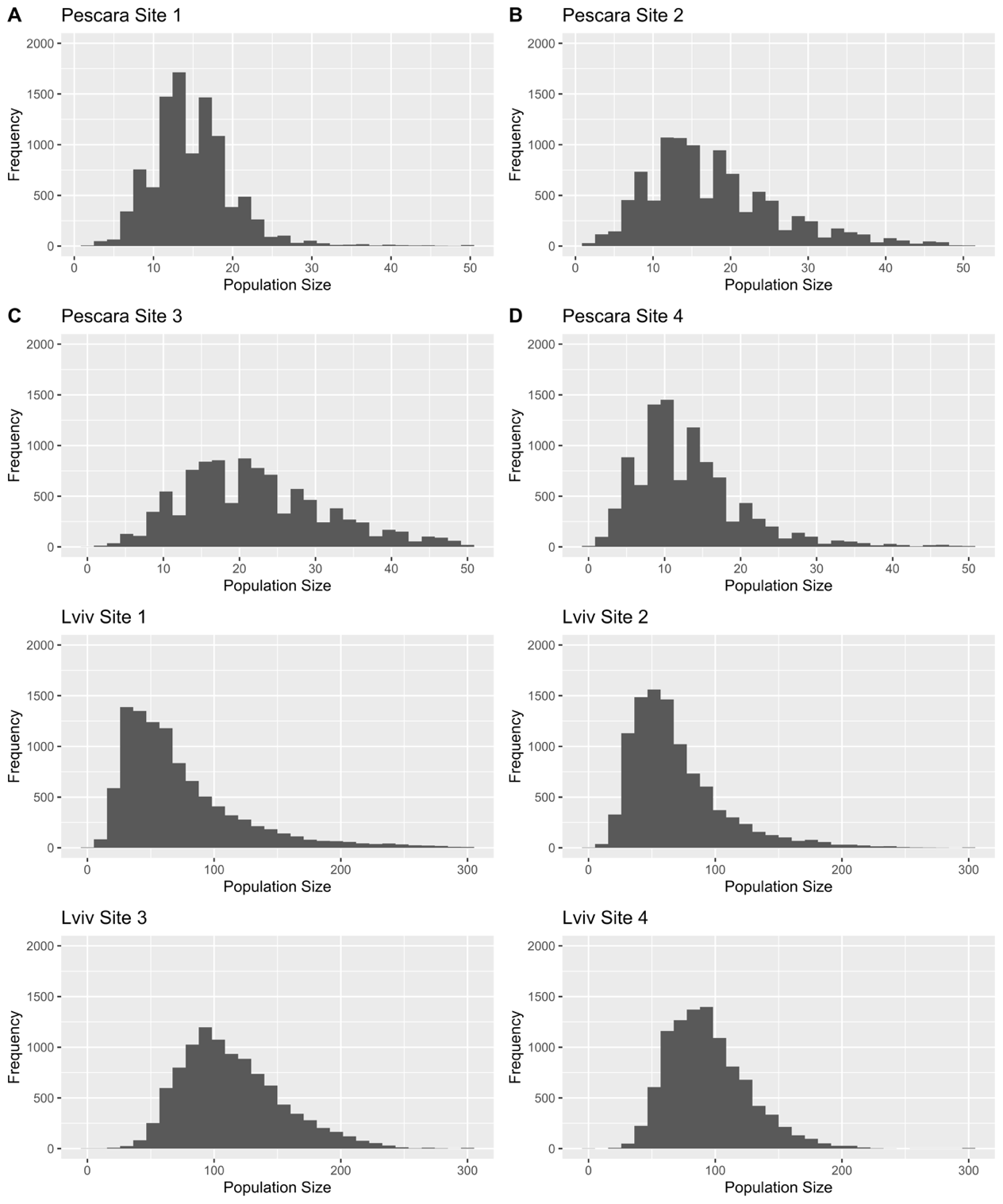

Supplement: S5 Fig — (TIF) [file pone.0266636.s005.tif]

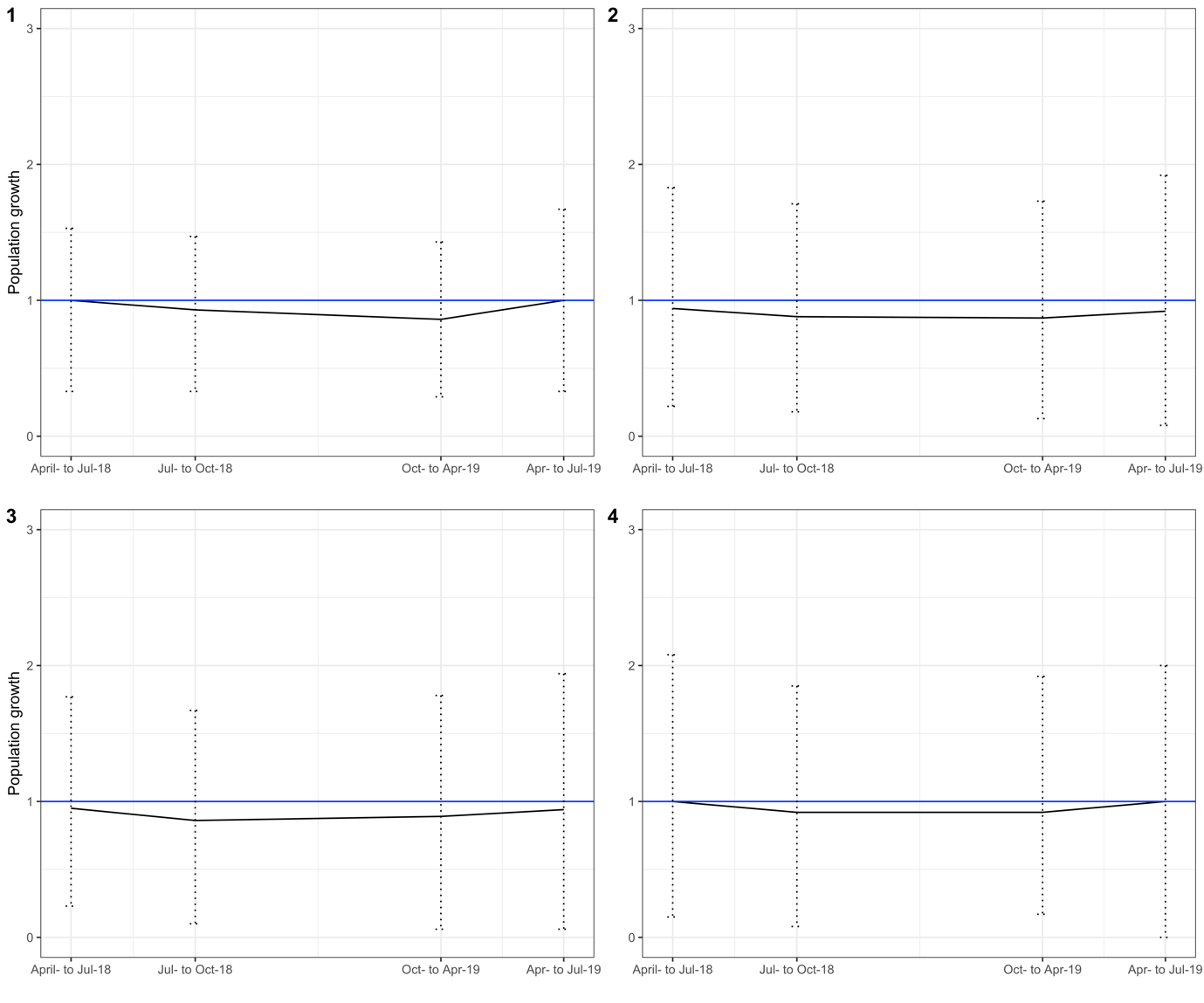

Supplement: S6 Fig — Error bars show the 2.5 and 97.5% limits of the highest posterior density credible intervals (CI) of the posterior distribution. Blue lines indicate stable population (i.e. no growth or decline). *Note uneven spacing as no surveys conducted in January 2019. (TIF) [file pone.0266636.s006.tif]

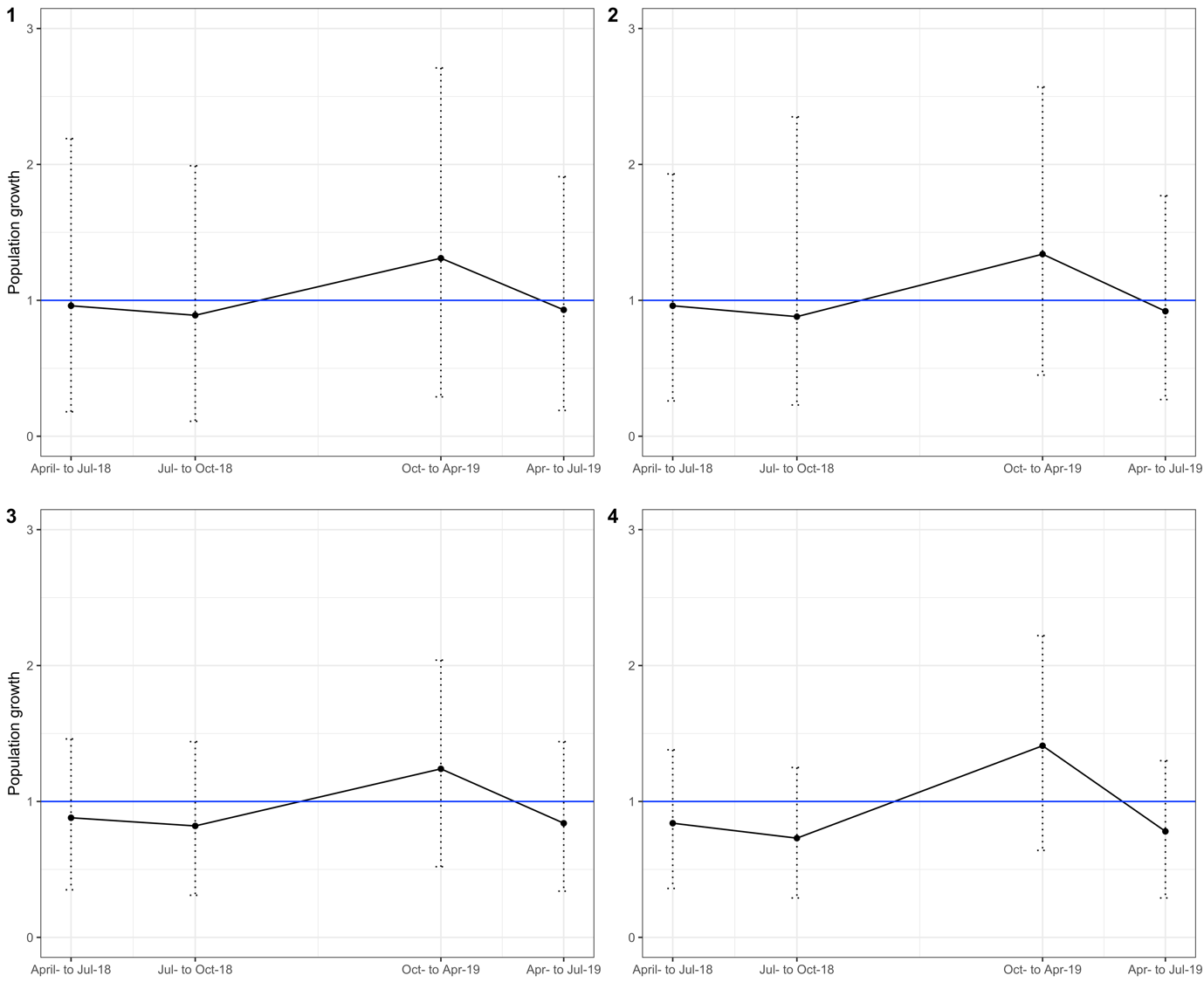

Supplement: S7 Fig — Error bars show the 2.5 and 97.5% limits of the highest posterior density credible intervals (CI) of the posterior distribution. Blue lines indicate stable population (i.e. no growth or decline).* Note uneven spacing as no surveys conducted in January 2019. (TIF) [file pone.0266636.s007.tif]
