# Supplementary material for: Population dynamics of free-roaming dogs in two European regions and implications for population control
Source: PLoS One. 2022 Sep 9;17(9):e0266636. doi: 10.1371/journal.pone.0266636 (PMC9462782; doi:10.1371/journal.pone.0266636)
Supplement: S1 Table — Sources: Veterinary Services–Pescara Province Local Health Unit for Pescara; and local Communal Enterprise for Lviv. (DOCX) [file pone.0266636.s008.docx]

**Supporting information – S1 Table**

**Population dynamics of free-roaming dogs and implications for population control**

Table S1. Numbers of dogs caught, neutered and released to study sites in Pescara, Italy and Lviv, Ukraine between 2014 and 2019.

Sources: Veterinary Services – Pescara Province Local Health Unit for Pescara; and local Communal Enterprise for Lviv.

|  |  | **Number of dogs released to study site** | | | | | | |
| --- | --- | --- | --- | --- | --- | --- | --- | --- |
|  | **Study site** | **2014** | **2015** | **2016** | **2017** | **2018** | **2019 (Jan-Jul)** | **Total** |
| Pescara | One | 5 | 11 | 7 | 13 | 4 | 4 | 44 |
|  | Two | 4 | 8 | 6 | 10 | 6 | 3 | 37 |
|  | Three | 3 | 8 | 6 | 3 | 4 | 10 | 34 |
|  | Four | 14 | 10 | 22 | 4 | 9 | 5 | 64 |
| Lviv | One | 0 | 0 | 69 | 105 | 89 | 7 | 270 |
|  | Two | 0 | 0 | 34 | 58 | 51 | 20 | 163 |
|  | Three | 0 | 0 | 0 | 0 | 0 | 0 | 0 |
|  | Four | 0 | 0 | 1 | 0 | 0 | 0 | 1 |
