# Supplementary material for: Population dynamics of free-roaming dogs in two European regions and implications for population control
Source: PLoS One. 2022 Sep 9;17(9):e0266636. doi: 10.1371/journal.pone.0266636 (PMC9462782; doi:10.1371/journal.pone.0266636)
Supplement: S2 Table — (DOCX) [file pone.0266636.s009.docx]

**Supporting information – S2 Table**

**Population dynamics of free-roaming dogs and implications for population control**

Table S2. Survey timings, distance and length (minimum, maximum and mean) in study sites in Pescara, Italy and Lviv, Ukraine.

| **Study region** | **Study site** | **Distance (km)** | **Survey time (minutes)** | | | **Start time** | | | **End time** | | |
| --- | --- | --- | --- | --- | --- | --- | --- | --- | --- | --- | --- |
|  |  |  | **Min** | **Max** | **Mean** | **Min** | **Max** | **Mean** | **Min** | **Max** | **Mean** |
| Pescara | One | 3.38 | 49 | 97 | 63 | 06:53 | 07:08 | 06:59 | 07:44 | 08:30 | 08:01 |
|  | Two | 6.20 | 49 | 81 | 67 | 06:55 | 07:09 | 07:00 | 07:49 | 08:17 | 08:07 |
|  | Three | 8.79 | 76 | 128 | 92 | 06:52 | 07:15 | 07:01 | 08:16 | 09:00 | 08:33 |
|  | Four | 6.50 | 77 | 106 | 87 | 06:00* | 07:21 | 06:57 | 07:18 | 08:45 | 08:24 |
| Lviv | One | 8.44 | 77 | 153 | 99 | 06:50 | 07:13** | 07:00 | 08:14 | 08:57 | 08:32 |
|  | Two | 7.20 | 86 | 112 | 95 | 06:45 | 07:30** | 07:01 | 08:13 | 09:01 | 08:36 |
|  | Three | 9.43 | 74 | 121 | 101 | 05:58* | 07:40** | 07:01 | 07:53 | 09:14 | 08:48 |
|  | Four | 7.64 | 86 | 135 | 107 | 06:50 | 07:31** | 07:05 | 08:26 | 09:27 | 08:51 |

* Survey began earlier due to logistical constraints.

** Survey began later due to daylight hours (sunrise at later time).
