# Supplementary material for: Population dynamics of free-roaming dogs in two European regions and implications for population control
Source: PLoS One. 2022 Sep 9;17(9):e0266636. doi: 10.1371/journal.pone.0266636 (PMC9462782; doi:10.1371/journal.pone.0266636)
Supplement: S3 Table — (DOCX) [file pone.0266636.s010.docx]

**Supporting information – S3 Table**

**Population dynamics of free-roaming dogs and implications for population control**

Table S3. Primary and secondary sampling period timings, temperature and weather conditions in Pescara, Italy.

| **Primary sampling period** | **Study site** | **Secondary sampling period** | **Date** | **Start Temp** | **Finish Temp** | **Mean temperature (^o^C)** | **Rain** | **Market event** | **Start time** | **Finish time** | **Survey length (minutes)** |
| --- | --- | --- | --- | --- | --- | --- | --- | --- | --- | --- | --- |
| One (April 2018) | One | 1 | 11/04/2018 | 11 | 11 | 11 | Yes | No | 07:00 | 07:57 | 57 |
|  |  | 2 | 12/04/2018 | 13 | 13 | 13 | No | No | 07:00 | 08:01 | 61 |
|  |  | 3 | 13/04/2018 | 11 | 12 | 11.5 | No | No | 06:53 | 08:30 | 97 |
|  | Two | 1 | 14/04/2018 | 11 | 12 | 11.5 | No | No | 06:55 | 08:16 | 81 |
|  |  | 2 | 15/04/2018 | 13 | 13 | 13 | No | No | 06:55 | 08:16 | 81 |
|  |  | 3 | 16/04/2018 | 14 | 14 | 14 | No | No | 07:00 | 08:09 | 69 |
|  | Three | 1 | 17/04/2018 | 12 | 13 | 12.5 | No | No | 06:52 | 08:26 | 94 |
|  |  | 2 | 18/04/2018 | 14 | 14 | 14 | No | No | 06:58 | 08:42 | 105 |
|  |  | 3 | 19/04/2018 | 13 | 16 | 14.5 | No | No | 06:52 | 09:00 | 128 |
|  | Four | 1 | 20/04/2018 | 14 | 17 | 15.5 | No | No | 06:55 | 08:41 | 106 |
|  |  | 2 | 21/04/2018 | 14 | 14 | 14 | No | No | 06:49 | 08:23 | 94 |
|  |  | 3 | 22/04/2018 | 12 | 14 | 13 | No | No | 06:50 | 08:16 | 86 |
| Two (July 2018) | One | 1 | 06/07/2018 | 22 | 23 | 22.5 | No | No | 06:55 | 08:03 | 68 |
|  |  | 2 | 07/07/2018 | 20 | 22 | 21 | No | No | 07:00 | 08:20 | 80 |
|  |  | 3 | 08/07/2018 | 20 | 22 | 21 | No | No | 06:55 | 08:10 | 75 |
|  | Two | 1 | 09/07/2018 | 21 | 24 | 22.5 | No | No | 07:03 | 08:10 | 67 |
|  |  | 2 | 10/07/2018 | 19 | 22 | 20.5 | No | No | 06:55 | 08:12 | 77 |
|  |  | 3 | 11/07/2018 | 24 | 24 | 24 | No | No | 07:00 | 08:00 | 60 |
|  | Three | 1 | 12/07/2018 | 20 | 23 | 21.5 | No | No | 07:09 | 08:57 | 108 |
|  |  | 2 | 13/07/2018 | 22 | 24 | 23 | No | No | 07:00 | 08:34 | 94 |
|  |  | 3 | 14/07/2018 | 21 | 24 | 22.5 | No | Yes | 07:06 | 08:36 | 90 |
|  | Four | 1 | 15/07/2018 | 23 | 26 | 24.5 | No | No | 07:05 | 08:39 | 94 |
|  |  | 2 | 16/07/2018 | 23 | 26 | 24.5 | No | No | 07:05 | 08:33 | 88 |
|  |  | 3 | 17/07/2018 | 19 | 19 | 19 | Yes | No | 07:01 | 08:20 | 79 |
| Three (October 2018) | One | 1 | 02/10/2018 | 12 | 13 | 12.5 | No | No | 07:00 | 08:00 | 60 |
|  |  | 2 | 03/10/2018 | 12 | 13 | 12.5 | No | No | 07:00 | 08:00 | 60 |
|  |  | 3 | 04/10/2018 | 13 | 14 | 13.5 | No | No | 06:55 | 08:02 | 67 |
|  | Two | 1 | 05/10/2018 | 16 | 15 | 15.5 | Yes | Yes | 07:06 | 08:09 | 63 |
|  |  | 2 | 06/10/2018 | 14 | 16 | 15 | Yes | No | 07:01 | 08:17 | 76 |
|  |  | 3 | 07/10/2018 | 14 | 16 | 15 | No | No | 06:58 | 08:10 | 72 |
|  | Three | 1 | 08/10/2018 | 13 | 14.5 | 13.75 | No | No | 07:00 | 08:32 | 92 |
|  |  | 2 | 09/10/2018 | 14 | 15 | 14.5 | No | No | 07:02 | 08:34 | 92 |
|  |  | 3 | 10/10/2018 | 14 | 15 | 14.5 | No | No | 07:15 | 08:45 | 90 |
|  | Four | 1 | 11/10/2018 | 14 | 16 | 15 | No | No | 07:15 | 08:45 | 90 |
|  |  | 2 | 12/10/2018 | 17 | 17 | 17 | No | No | 06:56 | 08:22 | 86 |
|  |  | 3 | 13/10/2018 | 13 | 14 | 13.5 | No | No | 07:21 | 08:43 | 82 |
| Four (April 2019) | One | 1 | 07/04/2019 | 8 | 9 | 8.5 | Yes | No | 07:00 | 07:51 | 51 |
|  |  | 2 | 08/04/2019 | 10 | 9 | 9.5 | No | No | 07:08 | 08:04 | 56 |
|  |  | 3 | 09/04/2019 | 12 | 12 | 12 | No | No | 06:55 | 07:44 | 49 |
|  | Two | 1 | 10/04/2019 | 9 | 10 | 9.5 | No | No | 07:00 | 08:03 | 63 |
|  |  | 2 | 11/04/2019 | 7 | 9 | 8 | No | No | 07:09 | 08:07 | 58 |
|  |  | 3 | 12/04/2019 | 7 | 9 | 8 | No | No | 07:00 | 07:49 | 49 |
|  | Three | 1 | 13/04/2019 | 7 | 7 | 7 | No | Yes | 07:00 | 08:23 | 83 |
|  |  | 2 | 14/04/2019 | 7 | 8 | 7.5 | No | No | 07:00 | 08:23 | 83 |
|  |  | 3 | 15/04/2019 | 7 | 7 | 7 | Yes | No | 07:00 | 08:22 | 82 |
|  | Four | 1 | 16/04/2019 | 9 | 10 | 9.5 | No | No | 07:00 | 08:20 | 80 |
|  |  | 2 | 17/04/2019 | 9 | 12 | 10.5 | No | Yes | 07:00 | 08:23 | 83 |
|  |  | 3 | 18/04/2019 | 11 | 12 | 11.5 | No | No | 07:00 | 08:44 | 104 |
| Five (July 2019) | One | 1 | 09/07/2019 | 25 | 26 | 25.5 | No | No | 07:05 | 08:04 | 59 |
|  |  | 2 | 10/07/2019 | 23 | 23 | 23 | No | No | 07:00 | 07:52 | 52 |
|  |  | 3 | 11/07/2019 | 19 | 21 | 20 | No | Yes | 07:00 | 07:50 | 50 |
|  | Two | 1 | 12/07/2019 | 19 | 22 | 20.5 | No | Yes | 06:57 | 08:06 | 69 |
|  |  | 2 | 13/07/2019 | 20 | 22 | 21 | No | No | 07:03 | 08:00 | 57 |
|  |  | 3 | 14/07/2019 | 18 | 19 | 18.5 | No | No | 07:05 | 08:02 | 57 |
|  | Three | 1 | 15/07/2019 | 17 | 19 | 18 | No | No | 07:14 | 08:33 | 79 |
|  |  | 2 | 16/07/2019 | 17 | 18 | 17.5 | No | No | 07:00 | 08:16 | 76 |
|  |  | 3 | 17/07/2019 | 17 | 19 | 18 | No | No | 07:01 | 08:26 | 85 |
|  | Four | 1 | 18/07/2019 | 18 | 22 | 20 | No | No | 07:00 | 08:20 | 80 |
|  |  | 2 | 19/07/2019 | 19 | 21 | 20 | No | No | 07:00 | 08:17 | 77 |
|  |  | 3 | 20/07/2019 | 19 | 22 | 20.5 | No | No | 06:00 | 07:18 | 78 |
