# Supplementary material for: Population dynamics of free-roaming dogs in two European regions and implications for population control
Source: PLoS One. 2022 Sep 9;17(9):e0266636. doi: 10.1371/journal.pone.0266636 (PMC9462782; doi:10.1371/journal.pone.0266636)
Supplement: S4 Table — (DOCX) [file pone.0266636.s011.docx]

**Supporting information – S4 Table**

**Population dynamics of free-roaming dogs and implications for population control**

Table S4. Primary and secondary sampling period timings, temperature and weather conditions in Lviv, Ukraine.

| **Primary sampling period** | **Study site** | **Secondary sampling period** | **Date** | **Start Temp** | **Finish Temp** | **Mean Temperature (^o^C)** | **Rain** | **Market event** | **Start time** | **Finish time** | **Survey length (minutes)** |
| --- | --- | --- | --- | --- | --- | --- | --- | --- | --- | --- | --- |
| One (April 2018) | One | 1 | 01/05/2018 | 12 | 19 | 15.5 | No | No | 07:00 | 08:57 | 117 |
|  |  | 2 | 02/05/2018 | 11 | 17 | 14 | No | No | 06:55 | 08:53 | 118 |
|  |  | 3 | 03/05/2018 | 14 | 17 | 15.5 | No | No | 06:50 | 08:29 | 99 |
|  | Two | 1 | 04/05/2018 | 10 | 17 | 13.5 | No | No | 06:50 | 08:42 | 112 |
|  |  | 2 | 05/05/2018 | 13 | 14 | 13.5 | No | No | 06:45 | 08:21 | 96 |
|  |  | 3 | 06/05/2018 | 11 | 11 | 11 | No | No | 06:45 | 08:13 | 88 |
|  | Three | 1 | 07/05/2018 | 6 | 24 | 15 | No | No | 06:55 | 08:55 | 120 |
|  |  | 2 | 08/05/2018 | 11 | 13 | 12 | No | No | 06:55 | 08:50 | 115 |
|  |  | 3 | 09/05/2018 | 14 | 17 | 15.5 | No | No | 06:50 | 08:45 | 85 |
|  | Four | 1 | 10/05/2018 | 12 | 17 | 14.5 | No | No | 06:55 | 08:50 | 115 |
|  |  | 2 | 11/05/2018 | 10 | 16 | 13 | No | No | 06:55 | 08:26 | 91 |
|  |  | 3 | 12/05/2018 | 9 | 13 | 11 | No | No | 06:50 | 08:34 | 104 |
| Two (July 2018) | One | 1 | 20/07/2018 | 18 | 19 | 18.5 | No | No | 07:00 | 08:28 | 88 |
|  |  | 2 | 21/07/2018 | 17 | 19 | 18 | No | Yes | 07:00 | 08:47 | 107 |
|  |  | 3 | 22/07/2018 | 14 | 18 | 16 | No | No | 07:00 | 08:24 | 84 |
|  | Two | 1 | 23/07/2018 | 18 | 19 | 18.5 | No | No | 07:00 | 08:41 | 101 |
|  |  | 2 | 24/07/2018 | 18 | 19 | 18.5 | Yes | No | 07:00 | 08:36 | 96 |
|  |  | 3 | 25/07/2018 | 18 | 18 | 18 | No | No | 07:00 | 08:42 | 102 |
|  | Three | 1 | 30/07/2018 | 17 | 21 | 19 | No | No | 07:00 | 09:14 | 74 |
|  |  | 2 | 31/07/2018 | 18 | 19 | 18.5 | Yes | No | 07:00 | 08:48 | 108 |
|  |  | 3 | 01/08/2018 | 18 | 19 | 18.5 | No | No | 05:58 | 07:53 | 115 |
|  | Four | 1 | 27/07/2018 | 17 | 21 | 19 | No | No | 06:50 | 09:05 | 135 |
|  |  | 2 | 28/07/2018 | 17 | 21 | 19 | No | No | 06:55 | 08:58 | 124 |
|  |  | 3 | 29/07/2018 | 20 | 21 | 20.5 | No | No | 07:00 | 09:07 | 127 |
| Three (October 2018) | One | 1 | 16/10/2018 | 6 | 7 | 6.5 | No | No | 06:55 | 08:28 | 94 |
|  |  | 2 | 17/10/2018* | 5 | 7 | 6 | NA | NA | NA | NA | NA |
|  |  | 3 | 18/10/2018 | 7 | 7 | 7 | No | No | 07:13 | 08:46 | 153 |
|  | Two | 1 | 19/10/2018 | 7 | 9 | 8 | No | No | 07:20 | 08:50 | 90 |
|  |  | 2 | 20/10/2018 | 8 | 9 | 8.5 | No | No | 07:30 | 09:01 | 91 |
|  |  | 3 | 21/10/2018 | 7 | 7 | 7 | Yes | No | 07:28 | 09:00 | 92 |
|  | Three | 1 | 22/10/2018 | 0 | 1 | 0.5 | No | No | 07:27 | 09:07 | 100 |
|  |  | 2 | 23/10/2018 | 6 | 6 | 6 | Yes | No | 07:30 | 09:07 | 97 |
|  |  | 3 | 24/10/2018 | 6 | 6 | 6 | Yes | No | 07:40 | 09:11 | 91 |
|  | Four | 1 | 25/10/2018 | 3 | 3 | 3 | No | No | 07:31 | 09:27 | 122 |
|  |  | 2 | 26/10/2018 | 6 | 6 | 6 | No | No | 07:30 | 09:08 | 98 |
|  |  | 3 | 27/10/2018 | 8 | 8 | 8 | No | Yes | 07:30 | 09:13 | 103 |
| Four (April 2019) | One | 1 | 26/04/2019 | 9 | 15 | 12 | No | Yes | 07:00 | 08:36 | 94 |
|  |  | 2 | 27/04/2019 | 16 | 17 | 16.5 | No | No | 07:00 | 08:25 | 85 |
|  |  | 3 | 28/04/2019 | 12 | 12 | 12 | No | No | 06:57 | 08:14 | 77 |
|  | Two | 1 | 29/04/2019 | 9 | 10 | 9.5 | Yes | No | 07:00 | 08:26 | 86 |
|  |  | 2 | 30/04/2019 | 9 | 10 | 9.5 | Yes | No | 06:50 | 08:21 | 91 |
|  |  | 3 | 01/05/2019 | 8 | 8 | 8 | Yes | No | 06:55 | 08:24 | 89 |
|  | Three | 1 | 02/05/2019 | 7 | 10 | 8.5 | No | No | 06:57 | 08:58 | 121 |
|  |  | 2 | 03/05/2019 | 9 | 12 | 10.5 | No | No | 07:08 | 08:50 | 102 |
|  |  | 3 | 04/05/2019 | 8 | 8 | 8 | No | No | 07:02 | 08:49 | 107 |
|  | Four | 1 | 05/05/2019 | 9 | 9 | 9 | Yes | No | 07:06 | 08:49 | 103 |
|  |  | 2 | 06/05/2019 | 5 | 5 | 5 | Yes | No | 07:02 | 08:51 | 109 |
|  |  | 3 | 07/05/2019 | 5 | 6 | 5.5 | No | No | 07:04 | 08:51 | 107 |
| Five (July 2019) | One | 1 | 21/07/2019 | 15 | 19 | 17 | No | No | 07:02 | 08:20 | 78 |
|  |  | 2 | 22/07/2019 | 16 | 17 | 16.5 | No | No | 07:05 | 08:28 | 83 |
|  |  | 3 | 23/07/2019 | 16 | 18 | 17 | No | No | 07:00 | 08:18 | 78 |
|  | Two | 1 | 24/07/2019 | 16 | 17 | 16.5 | No | No | 07:00 | 08:32 | 92 |
|  |  | 2 | 25/07/2019 | 16 | 17 | 16.5 | No | No | 07:00 | 08:36 | 96 |
|  |  | 3 | 26/07/2019 | 16 | 18 | 17 | No | No | 07:02 | 08:44 | 102 |
|  | Three | 1 | 27/07/2019 | 14 | 17 | 15.5 | No | No | 07:01 | 08:38 | 97 |
|  |  | 2 | 28/07/2019 | 20 | 21 | 20.5 | No | No | 07:00 | 08:27 | 87 |
|  |  | 3 | 29/07/2019 | 19 | 23 | 21 | No | No | 07:00 | 08:35 | 95 |
|  | Four | 1 | 30/07/2019 | 18 | 19 | 18.5 | No | No | 07:03 | 08:32 | 89 |
|  |  | 2 | 31/07/2019 | 18 | 19 | 18.5 | No | No | 07:00 | 08:26 | 86 |
|  |  | 3 | 01/08/2019 | 16 | 18 | 17 | No | No | 07:07 | 08:33 | 86 |

***** Primary sampling period three, secondary sampling period two was missed due to fieldworker illness
