# Supplementary material for: Population dynamics of free-roaming dogs in two European regions and implications for population control
Source: PLoS One. 2022 Sep 9;17(9):e0266636. doi: 10.1371/journal.pone.0266636 (PMC9462782; doi:10.1371/journal.pone.0266636)
Supplement: S5 Table — (DOCX) [file pone.0266636.s012.docx]

**Supporting information – S5 Table**

**Population dynamics of free-roaming dogs and implications for population control**

Table S5. Probability of apparent survival and detection for primary sampling periods (averaged across individuals and study sites) and study sites (averaged across individuals and primary periods) in Pescara, Italy.

|  |  | **Mean** | **2.5% CI** | **97.5% CI** |
| --- | --- | --- | --- | --- |
| Average probability of apparent survival | Primary Period 1 to 2 (3-month interval) | 0.82 | 0.58 | 1.00 |
|  | Primary Period 2 to 3 (3-month interval) | 0.74 | 0.44 | 0.99 |
|  | Primary Period 3 to 4 (6-month interval) | 0.74 | 0.43 | 1.00 |
|  | Primary Period 4 to 5 (3-month interval) | 0.79 | 0.47 | 1.00 |
|  | study site 1 | 0.77 | 0.48 | 0.98 |
|  | study site 2 | 0.71 | 0.36 | 0.99 |
|  | study site 3 | 0.71 | 0.33 | 1.00 |
|  | study site 4 | 0.77 | 0.45 | 1.00 |
| Average probability of detecting a dog | Primary Period 1 | 0.23 | 0.04 | 0.47 |
|  | Primary Period 2 | 0.18 | 0.02 | 0.40 |
|  | Primary Period 3 | 0.20 | 0.03 | 0.42 |
|  | Primary Period 4 | 0.18 | 0.02 | 0.40 |
|  | Primary Period 5 | 0.25 | 0.04 | 0.52 |
|  | study site 1 | 0.41 | 0.14 | 0.74 |
|  | study site 2 | 0.18 | 0.01 | 0.46 |
|  | study site 3 | 0.11 | 0.01 | 0.27 |
|  | study site 4 | 0.22 | 0.01 | 0.51 |
