# Supplementary material for: Population dynamics of free-roaming dogs in two European regions and implications for population control
Source: PLoS One. 2022 Sep 9;17(9):e0266636. doi: 10.1371/journal.pone.0266636 (PMC9462782; doi:10.1371/journal.pone.0266636)
Supplement: S6 Table — (DOCX) [file pone.0266636.s013.docx]

**Supporting information – S6 Table**

**Population dynamics of free-roaming dogs and implications for population control**

Table S6. Probability of apparent survival and detection for primary sampling periods (averaged across individuals and study sites) and study sites (averaged across individuals and primary periods) in Lviv, Ukraine.

|  |  | **Mean** | **2.5% CI** | **97.5% CI** |
| --- | --- | --- | --- | --- |
| Average probability of apparent survival | Primary Period 1 to 2 (3-month interval) | 0.83 | 0.61 | 1.00 |
|  | Primary Period 2 to 3(3-month interval) | 0.76 | 0.50 | 0.97 |
|  | Primary Period 3 to 4 (6-month interval) | 0.90 | 0.73 | 1.00 |
|  | Primary Period 4 to 5(3-month interval) | 0.73 | 0.44 | 0.97 |
|  | study site 1 | 0.83 | 0.56 | 1.00 |
|  | study site 2 | 0.82 | 0.57 | 1.00 |
|  | study site 3 | 0.75 | 0.46 | 0.98 |
|  | study site 4 | 0.67 | 0.35 | 0.93 |
| Average probability of detecting a dog | Primary Period 1 | 0.08 | 0.00 | 0.21 |
|  | Primary Period 2 | 0.12 | 0.01 | 0.30 |
|  | Primary Period 3 | 0.14 | 0.01 | 0.34 |
|  | Primary Period 4 | 0.10 | 0.01 | 0.26 |
|  | Primary Period 5 | 0.10 | 0.01 | 0.25 |
|  | study site 1 | 0.07 | 0.00 | 0.22 |
|  | study site 2 | 0.11 | 0.00 | 0.31 |
|  | study site 3 | 0.10 | 0.00 | 0.28 |
|  | study site 4 | 0.16 | 0.01 | 0.41 |
