# Supplementary material for: Population dynamics of free-roaming dogs in two European regions and implications for population control
Source: PLoS One. 2022 Sep 9;17(9):e0266636. doi: 10.1371/journal.pone.0266636 (PMC9462782; doi:10.1371/journal.pone.0266636)
Supplement: S7 Table — (DOCX) [file pone.0266636.s014.docx]

**Supporting information – S7 Table**

**Population dynamics of free-roaming dogs and implications for population control**

Table S7. Standard deviations for between-dog effects on survival and detection on log odds scale.

|  |  | **Pescara** | | | **Lviv** | | |
| --- | --- | --- | --- | --- | --- | --- | --- |
|  | **Study site** | **Mean** | **2.5% CI** | **97.5% CI** | **Mean** | **2.5% CI** | **97.5% CI** |
| Survival (*φ*) | 1 | 0.95 | 0.00 | 2.14 | 1.02 | 0.00 | 2.31 |
|  | 2 | 1.01 | 0.00 | 2.30 | 0.77 | 0.00 | 1.87 |
|  | 3 | 0.85 | 0.00 | 2.06 | 1.22 | 0.00 | 2.47 |
|  | 4 | 0.78 | 0.00 | 1.92 | 0.63 | 0.00 | 1.54 |
| Detection (*δ*) | 1 | 0.55 | 0.00 | 1.37 | 1.58 | 0.81 | 2.34 |
|  | 2 | 1.47 | 0.45 | 2.44 | 1.54 | 0.81 | 2.31 |
|  | 3 | 0.48 | 0.00 | 1.17 | 1.85 | 1.20 | 2.49 |
|  | 4 | 1.05 | 0.04 | 2.00 | 1.67 | 1.01 | 2.31 |
