# Supplementary material for: Population dynamics of free-roaming dogs in two European regions and implications for population control
Source: PLoS One. 2022 Sep 9;17(9):e0266636. doi: 10.1371/journal.pone.0266636 (PMC9462782; doi:10.1371/journal.pone.0266636)
Supplement: S8 Table — (DOCX) [file pone.0266636.s015.docx]

**Supporting information – S8 Table**

**Population dynamics of free-roaming dogs and implications for population control**

Table S8. Comparison of mean apparent survival and detection as odds ratios between different study sites in Pescara, Italy and Lviv, Ukraine.

|  |  | Pescara | | | Lviv | | |
| --- | --- | --- | --- | --- | --- | --- | --- |
| Average probability | Study sites | Mean | 2.5% CI | 97.5% CI | Mean | 2.5% CI | 97.5% CI |
| Apparent survival (*φ*) | 1 and 2 | 2.19 | 0.01 | 6.50 | 2.11 | 0.02 | 6.52 |
|  | 1 and 3 | 2.62 | 0.01 | 7.97 | 3.41 | 0.06 | 10.76 |
|  | 1 and 4 | 1.44 | 0.01 | 4.06 | 5.69 | 0.12 | 17.36 |
|  | 2 and 3 | 2.08 | 0.00 | 6.79 | 2.56 | 0.07 | 6.98 |
|  | 2 and 4 | 1.31 | 0.00 | 3.95 | 4.20 | 0.33 | 11.21 |
|  | 3 and 4 | 1.54 | 0.00 | 4.77 | 2.08 | 0.23 | 4.89 |
| Detection (*δ*) | 1 and 2 | 6.68 | 0.18 | 20.69 | 0.87 | 0.01 | 2.76 |
|  | 1 and 3 | 9.26 | 0.55 | 21.59 | 0.92 | 0.01 | 2.88 |
|  | 1 and 4 | 4.57 | 0.14 | 14.13 | 0.48 | 0.01 | 1.47 |
|  | 2 and 3 | 2.72 | 0.05 | 8.40 | 1.65 | 0.03 | 4.94 |
|  | 2 and 4 | 1.30 | 0.01 | 4.38 | 0.84 | 0.01 | 2.34 |
|  | 3 and 4 | 0.65 | 0.03 | 2.05 | 0.71 | 0.05 | 1.85 |
