# Supplementary material for: Population dynamics of free-roaming dogs in two European regions and implications for population control
Source: PLoS One. 2022 Sep 9;17(9):e0266636. doi: 10.1371/journal.pone.0266636 (PMC9462782; doi:10.1371/journal.pone.0266636)
Supplement: S9 Table — (DOCX) [file pone.0266636.s016.docx]

**Supporting information – S9 Table**

**Population dynamics of free-roaming dogs and implications for population control**

Table S9. Comparison of mean apparent survival and detection as odds ratios between different intervals between primary periods in Pescara, Italy and Lviv, Ukraine.

|  |  | Pescara | | | Lviv | | |
| --- | --- | --- | --- | --- | --- | --- | --- |
| Average probability | Primary period | Mean | 2.5% CI | 97.5% CI | Mean | 2.5% CI | 97.5% CI |
| Apparent survival (*φ*) | 2 to 3 | 4.06 | 0.01 | 11.64 | 2.56 | 0.14 | 6.82 |
|  | 2 to 4 | 3.75 | 0.01 | 11.50 | 0.80 | 0.01 | 2.11 |
|  | 2 to 5 | 3.12 | 0.00 | 8.37 | 3.05 | 0.07 | 8.38 |
|  | 3 to 4 | 1.88 | 0.00 | 5.51 | 0.42 | 0.00 | 1.19 |
|  | 3 to 5 | 1.33 | 0.00 | 4.16 | 1.54 | 0.09 | 4.00 |
|  | 4 to 5 | 1.49 | 0.00 | 4.36 | 7.81 | 0.16 | 24.16 |
| Detection (*δ*) | 1 to 2 | 1.70 | 0.31 | 3.71 | 0.65 | 0.29 | 1.07 |
|  | 1 to 3 | 1.29 | 0.46 | 2.37 | 0.59 | 0.19 | 1.10 |
|  | 1 to 4 | 1.59 | 0.47 | 3.09 | 0.80 | 0.31 | 1.39 |
|  | 1 to 5 | 1.10 | 0.20 | 2.40 | 0.87 | 0.31 | 1.56 |
|  | 2 to 3 | 0.93 | 0.21 | 1.89 | 0.96 | 0.28 | 1.79 |
|  | 2 to 4 | 1.26 | 0.11 | 3.13 | 1.31 | 0.53 | 2.28 |
|  | 2 to 5 | 0.71 | 0.23 | 1.34 | 1.38 | 0.66 | 2.27 |
|  | 3 to 4 | 1.33 | 0.41 | 2.50 | 1.45 | 0.80 | 2.26 |
|  | 3 to 5 | 0.89 | 0.20 | 1.76 | 1.66 | 0.50 | 3.19 |
|  | 4 to 5 | 0.79 | 0.09 | 1.78 | 1.14 | 0.45 | 1.96 |
