# Supplementary material for: Population dynamics of free-roaming dogs in two European regions and implications for population control
Source: PLoS One. 2022 Sep 9;17(9):e0266636. doi: 10.1371/journal.pone.0266636 (PMC9462782; doi:10.1371/journal.pone.0266636)
Supplement: S1 File — (DOCX) [file pone.0266636.s017.docx]

**Supporting information – S1 File**

**Population dynamics of free-roaming dogs and implications for population control**

L. M. Smith^1^, C. Goold^1^, R. J. Quinnell^1^, A.M. Munteanu^2^, S. Hartmann^2^, P. Dalla Villa^3,4^, L. M. Collins*^1^

^1.^ Faculty of Biological Sciences, University of Leeds, Leeds, UK

^2.^ VIER PFOTEN International, Vienna, Austria

^3.^ Istituto Zooprofilattico Sperimentale dell’Abruzzo e del Molise “G. Caporale”, Teramo, Italy

^4.^ World Organization for Animal Health, OIE Sub-Regional Representation in Brussels, Belgium

# Survey timings

All surveys took place between 06:00 and 10:00. In Ukraine, out of 60 surveys, 58 (97%) surveys took place between 06:30 and 09:30, one survey (2%) was missed due to illness, and one survey (2%) began at 06:00 due to logistical constraints. In Italy, out of the 60 surveys, 59 (98%) took place between 06:30 and 09:30, and one survey began at 06:00 due to logistical constraints. See S2 table for more details.
