# Supplementary material for: Population dynamics of free-roaming dogs in two European regions and implications for population control
Source: PLoS One. 2022 Sep 9;17(9):e0266636. doi: 10.1371/journal.pone.0266636 (PMC9462782; doi:10.1371/journal.pone.0266636)
Supplement: S2 File — (DOCX) [file pone.0266636.s018.docx]

**Supporting information – S3 File**

**Population dynamics of free-roaming dogs and implications for population control**

L. M. Smith^1^, C. Goold^1^, R. J. Quinnell^1^, A.M. Munteanu^2^, S. Hartmann^2^, P. Dalla Villa^3,4^, L. M. Collins*^1^

^1.^ Faculty of Biological Sciences, University of Leeds, Leeds, UK

^2.^ VIER PFOTEN International, Vienna, Austria

^3.^ Istituto Zooprofilattico Sperimentale dell’Abruzzo e del Molise “G. Caporale”, Teramo, Italy

^4.^ World Organization for Animal Health, OIE Sub-Regional Representation in Brussels, Belgium

**Details of hierarchical Bayesian hidden Markov model of Pollock’s robust design**

Parameter-expansion and data augmentation:

Parameter-expansion and data augmentation simply involved adding a list of all-zero capture histories to the data to account for individuals that were never observed over the duration of the mark-recapture study. This allowed the states of both the individuals that were observed and those that were unobserved throughout the study (i.e. those that had very low detection probabilities) to be modelled, allowing better inferences to be made about the true population (Kery & Schaub, 2011; Rankin et al., 2016; Royle & Dorazio, 2008).

Specifically, parameter expanded data-augmentation deals with the computational challenges of variable dimension space when modelling full-capture histories and random effects for individual dogs (Kery & Schaub, 2011; Rankin et al., 2016; Royle & Dorazio, 2012; Tanner & Wong, 1987). In this study, a set of *pseudo-individuals* with all-zero (*unobserved*) capture histories were included in the list of capture histories for each of the study sites. The augmented dataset (*m*) totalled 150 individuals at each primary period in Pescara and 300 individuals at each primary period in Lviv. The augmented dataset (*m*) included the observed number of individuals (*n*) plus a number of *pseudo-individuals*, and the estimated number of individuals (*N*) lies between *n* and *m*. The *pseudo-individuals* did not affect the estimates of detection probability (*δ*), apparent survival (*φ*) or population size (*N*) but allowed more accurate estimation of the parameters using simpler computation. To test that the dataset included enough *pseudo-individuals*, the posterior distributions of *N* were plotted to ensure the distribution was not truncated to the right (S1 to S5 Figures). The uncaptured *pseudo-individuals* made up the population of individuals that were available for recruitment into the study population and allowed modelling of individual random effects for dogs that were missed throughout all secondary sampling periods.

## Entry probability

This provided the fraction of the true population (‘super-population’; total number of dogs that had ever been in the study site across all primary periods) of individuals entering the study site at time *t*, given they had not entered at a previous time point. The entry probability must sum to one across all primary sampling periods and individuals were assumed to be in the *not yet entered* state prior to the first primary period. This means the entry probability calculated for the first primary period was less interpretable; instead, entry probabilities after the first primary period were reported. We also estimated a per capita entry probability (*f*), as described by Kery and Schaub (2011). Per capita entry probability describes the fraction of new recruits at primary period *t* per individual dog alive and in the study site at primary period *t*. This was calculated by Equation 1. Population growth (*l_t_*) was calculated by dividing the estimated population size at period *t* (*N_t_*) by the estimated population size at primary period *N_t-1_* (Equation 2). Table S5 outlines the parameters calculated for each study site.

Equation 1. Per capita entry probability.

$$f_{t}=\frac{E_{t}\times W}{N_{t}}$$

Equation 2. Population growth

$$\lambda_{t}= \frac{N_{t}}{N_{t-1}}$$

## Model running

Data from study sites in Pescara and Lviv were run in the same model, but parameter estimates were not informed by capture histories between countries (i.e. parameter estimates for study sites in Pescara were not informed by those estimated for study sites in Lviv).

**References**

Kery, M., & Schaub, M. (2011). Estimation of Survival, Recruitment, and Population Size from Capture-Recapture Data Using the Jolly-Seber Model. In *Bayesian Population Analysis using WinBUGS: A Hierarchical Perspective* (First, pp. 316–346). Waltham: Elsevier Inc.

Rankin, R. W., Nicholson, K. E., Allen, S. J., Krützen, M., Bejder, L., & Pollock, K. H. (2016). A Full-Capture Hierarchical Bayesian Model of Pollock’s Closed Robust Design and Application to Dolphins. *Frontiers in Marine Science*, *3*(25), 1–18. doi: 10.3389/fmars.2016.00025

Royle, J. A., & Dorazio, R. M. (2008). Modeling Population Dynamics. In A. J. Royle & R. M. Dozario (Eds.), *Hierarchical Modeling and Inference in Ecology: The analysis of data from populations, metapopulations and communities* (First, pp. 325–345). San Diego, United States: Elsevier Science Publishing Co Inc. doi: 10.1016/b978-0-12-374097-7.00012-0

Royle, J. A., & Dorazio, R. M. (2012). Parameter-expanded data augmentation for Bayesian analysis of capture-recapture models. *Journal of Ornithology*, *152*, 521–537. doi: https://doi.org/10.1007/s10336-010-0619-4

Tanner, M. A., & Wong, W. H. (1987). The calculation of posterior distributions by data augmentation. *Journal of the American Statistical Association*, *82*(398), 528–540. doi: 10.1080/01621459.1987.10478458
